# Effects of Trace Metal Concentrations on the Growth of the Coral Endosymbiont *Symbiodinium kawagutii*

**DOI:** 10.3389/fmicb.2016.00082

**Published:** 2016-02-08

**Authors:** Irene B. Rodriguez, Senjie Lin, Jiaxuan Ho, Tung-Yuan Ho

**Affiliations:** ^1^Research Center for Environmental Changes, Academia SinicaTaipei, Taiwan; ^2^State Key Laboratory of Marine Environmental Science, Xiamen UniversityXiamen, China; ^3^Department of Marine Sciences, University of Connecticut, GrotonCT, USA; ^4^School of Marine Sciences and Engineering, Plymouth UniversityDevon, UK

**Keywords:** trace metal limitation, coral bleaching, dinoflagellates, endosymbionts, zooxanthellae, oxidative stress

## Abstract

*Symbiodinium* is an indispensable endosymbiont in corals and the most important primary producer in coral reef ecosystems. During the past decades, coral bleaching attributed to the disruption of the symbiosis has frequently occurred resulting in reduction of coral reef coverage globally. Growth and proliferation of corals require some specific trace metals that are essential components of pertinent biochemical processes, such as in photosynthetic systems and electron transport chains. In addition, trace metals are vital in the survival of corals against oxidative stress because these metals serve as enzymatic cofactors in antioxidative defense mechanisms. The basic knowledge about trace metal requirements of *Symbiodinium* is lacking. Here we show that the requirement of *Symbiodinium kawagutii* for antioxidant-associated trace metals exhibits the following order: Fe >> Cu/Zn/Mn >> Ni. In growth media with Cu, Zn, Mn, and varying Fe concentrations, we observed that Cu, Zn, and Mn cellular quotas were inversely related to Fe concentrations. In the absence of Cu, Zn, and Mn, growth rates increased with increasing inorganic Fe concentrations up to 1250 pM, indicating the relatively high Fe requirement for *Symbiodinium* growth and potential functional complementarity of these metals. These results demonstrate the relative importance of trace metals to sustain *Symbiodinium* growth and a potential metal inter replacement strategy in *Symbiodinium* to ensure survival of coral reefs in an oligotrophic and stressful environment.

## Introduction

The dinoflagellate genus of *Symbiodinium* is widely considered as the most important constituent of coral holobionts (the coral and its collective community), because it generates photosynthetic products that are translocated to the coral host (Muscatine et al., 1981; Hoegh-Guldberg, 1999). In exchange for photosynthates, coral hosts and other members of holobionts provide *Symbiodinium* with inorganic carbon and other nutrients. The tight cycling and interaction of nutrients are crucial for survival of holobionts in an environment where dealing with multiple stressors, such as rapid changes in temperature, light intensity, and dissolved inorganic carbon concentration, is inevitable (Muscatine et al., 1981; Buck et al., 2002; Buxton et al., 2009; Downs et al., 2013). In recent years, widespread coral bleaching brought by temperature increase either independently or in synergy with other factors are becoming more of a norm (Fitt et al., 2001; Coles and Brown, 2003; Hughes et al., 2003; Hoegh-Guldberg et al., 2007). Coral bleaching is brought about by the decline or loss of *Symbiodinium* (commonly known as zooxanthellae due to its yellow–brown color) from the coral endoderm, hence resulting in discoloration (Lesser, 1996; Fitt et al., 2001; Coles and Brown, 2003). Additionally, the discoloration may consequently occur due to the loss of photosynthetic pigments thereby resulting in termination of the symbiotic association (Lesser, 1996; Fitt et al., 2001; Coles and Brown, 2003). The mechanism by which the cnidarian-dinoflagellate symbiosis is disrupted is a topic of contention, but there are various lines of evidence that this symbiotic relationship is disrupted by oxidative stress (Dykens and Shick, 1982; Lesser, 1996; Hoegh-Guldberg, 1999; Warner et al., 1999). In an environment constantly exposed to extreme conditions, both host and symbiont take part in dealing with stress by implementing various anti-oxidative defenses such as mycosporine-like amino acids and enzymatic antioxidants (Lesser and Shick, 1989; Levy et al., 2006; Roth, 2014). Amidst this suite of available photoprotective defenses, antioxidants are particularly important as coral reef ecosystems are subjected to high light intensity, high temperature, and even heavy metal contamination that are conducive to producing oxidative stress.

Trace metals are essential components of electron transport chains or important cofactors in enzymes involved in various biological processes, such as chlorophyll synthesis, nitrate reduction, and photoprotection or photorepair (Raven et al., 1999; Twining and Baines, 2013). Trace metals are also utilized in a suite of antioxidants, such as superoxide dismutases (SOD), catalase, and peroxidase. While all these anti-oxidative enzymes require Fe, SOD may also require Mn, Ni, or Cu/Zn as metal cofactors depending on the type of SOD (Matta et al., 1992; Dupont et al., 2008). The action of these antioxidative enzymes is necessary to maintain the cellular levels of reactive oxygen species (ROS). Previous *Symbiodinium* genomic studies have shown that some species contain all four types of SOD as part of their antioxidant gene repertoire (Matta et al., 1992; Bayer et al., 2012). Transcriptomics data generated recently indicate the presence of Fe/Mn-, Mn-, Cu/Zn-, and Ni-containing SOD in *Symbiodinium kawagutii* (GenBank accession number KC937072–KC951106; Zhang et al., 2013; Lin et al., 2015).

Trace metal requirements in other major marine phytoplankton have been intensively studied (e.g., Sunda, 2012) but their requirements in *Symbiodinium* remain unexplored. In this work, the major objective was to elucidate the roles of Fe and other trace metals on the growth of *Symbiodinium*. Because Fe is used in numerous biological processes we decided to focus first on the effect of Cu, Zn, Mn, and Ni on *S. kawagutii* growth, and then subsequently studied the effect of Fe in greater depth. The knowledge derived from this work lays the foundation for future research to better understand the biochemical processes requiring trace metals in *Symbiodinium*.

## Materials and Methods

### Culture Conditions

*Symbiodinium kawagutii* strain CCMP2468 (non-axenic but handled aseptically in this study) were grown in 500 ml polycarbonate bottles with trace-metal defined medium modified from the L1 medium recipe (Guillard and Hargraves, 1993). Surface seawater collected from the Western Philippine Sea (N23.5° E126°) was used for the medium preparation in all experiments. The seawater was filtered using Whatman^®^ Polycap filters, passed through a column packed with Chelex^®^ 100 resin to remove trace metal contents, and filter-sterilized using 0.22 μm pore size filters prior to use. The background trace metal concentrations in the seawater were determined by first pre-concentrating the metals using an automated flow injection-ion chromatograph pretreatment system, which is equipped with a Nobias Chelate-PA1 resin for selective retention of trace metals, prior to element detection in the eluate by high resolution-inductively coupled plasma mass spectrometer (HR-ICPMS). The concentrations were found to be 0.35, 2.0, 0.70, 0.56, and 2.0 nM for Fe, Mn, Zn, Cu, and Ni, respectively (Ho et al., 2010; Wang et al., 2014). Previous work has shown that passing natural or artificial seawater through a column packed with Chelex^®^ 100 resin substantially reduces the trace metal content, which allows us to control the trace metal concentrations as desired (Ho, 2013).

In all experiments, total initial concentration of phosphate and nitrate were 50 and 800 μM, respectively. Vitamin B mixture, composed of thiamine, biotin, and cyanocobalamin, was added so that final concentrations of B-vitamins in the medium were equivalent to 300, 2.05, and 0.4 nM, respectively. Stock solutions of trace metals and nutrients were prepared from analytical reagent-grade chemicals to minimize impurities. In addition, phosphate and nitrate standards were further purified by passing through Chelex^®^ 100 resin to remove metal impurities. All stock standards were filter-sterilized using 0.22 μm pore size filters. Bioavailability of the metals was controlled by adding ethylenediaminetetraacetic acid (EDTA) at 20 μM. All treatments were carried out in triplicates and all necessary procedures were performed in a class 100 trace-metal clean laboratory. The cultures were kept in growth chambers where light and dark periods were controlled under a square-wave 12:12 H light:dark regime. The growth chamber was set at either 23 or 27°C, and the photon irradiance was set at either 250 or 600 μE m^-2^ s^-1^. The light intensity was achieved by placing the culture bottles at appropriate distances from the light source and the intensity was validated by measuring the light penetration PAR using a submersible radiometer (Biospherical Instruments Inc. QSL 2100). Culture vessels and other materials used for culturing and related work were carefully washed with 2% Micro-90^®^ solution, rinsed, soaked with 10% hydrochloric acid solution, and rinsed thoroughly with ultrapure water prepared using a Milli-Q system.

### Influence of Cu, Zn, Mn, and Ni on *Symbiodinium* Growth

We carried out two major experiments in this study, involving the trace metals Cu, Zn, Mn, Ni, and Fe. These trace metals are of interest, because these are cofactors of different forms of SOD, the activity of which may be a significant factor in anti-oxidative defenses of host-*Symbiodinium* assemblages in the natural environment. The first major experiment was performed using equivalent Fe concentrations with specific treatments supplied with or without Cu/Zn, Mn, and Ni, individually or in pairs. Cu and Zn were bundled together because these two are both cofactors in Cu/Zn-SOD. Thus, results from treatments involving Cu and Zn mean that effects observed may be due to Cu or Zn alone or due to action of both. This experimental design led to a total of seven treatments including a control treatment, which included all the trace metals in the medium. The total dissolved Fe concentration used in all treatments was 500 nM resulting in Fe′ concentration of 2.5 nM. In this experiment, dissolved concentrations of trace metals were added at these total values: 100 nM for Mn, Ni, and Zn, and 10 nM for Cu. These resulted in inorganic metal (M′) concentrations of 42 nM, 67 pM, 125 pM, and 0.50 pM for Mn, Ni, Zn, and Cu, respectively (Westall et al., 1976). For treatments where a specific metal was proposed to be low, the metal of interest was not added to the culture medium. *S. kawagutii* were acclimatized to trace metal conditions in respective treatments for one transfer prior to final experiments. For the final experiment, 2 ml of culture was aseptically transferred to new culture medium, which resulted in 250 times dilution of carried over trace metals from the previous medium.

### Influence of Fe on *Symbiodinium* Growth

The second major experiment was carried out to better understand the effect of Fe availability on *Symbiodinium* growth. To achieve this, we designed two sets of treatments using varying Fe concentrations, wherein one set of treatments contained Cu, Zn, and Mn, and a second set without these three metals. In this major experiment, Fe concentration was varied such that different treatments have 0, 10, 50, 100, or 250 nM Fe, which resulted in Fe′ concentrations of 0, 50, 250, 500, or 1250 pM, respectively (Westall et al., 1976). The other metals were added to the medium, whenever necessitated by the research design, following the concentrations used in the first major experiment. This experimental design led to a total of 10 treatments. In this experiment, *S. kawagutii* were also acclimatized to the different trace metal concentrations. In the final experiments, however, some treatments with low Fe′ concentrations were inoculated using cells taken from cultures acclimatized to higher Fe′ conditions, because of low cell densities in the respective acclimatized cultures. In the set with Cu, Zn, and Mn, the treatment with 0 pM Fe′ was inoculated using cells acclimatized to 50 pM Fe′; in the set without Cu, Zn, and Mn, the treatments with 0 and 50 pM Fe′ were inoculated using cells acclimatized to 250 pM Fe′.

### Growth Monitoring and Intracellular Metal Quota Determination

The growth of *S. kawagutii* was monitored every other day by measuring cell density per ml using a Beckman Coulter Counter Multisizer 3 equipped with a 100 μm aperture tube until decline in biomass was observed. The growth rates and intracellular metal quotas were evaluated while the cultures were in the exponential phase of growth. The intracellular metal quotas were determined using cells harvested by filtration onto acid-washed polycarbonate filters (25 mm with 2 μm pore size) during the light phase of the light:dark regime. The filtered cells were washed with ultrapure water and digested using concentrated HNO_3_ prior to elemental analysis using HR-ICPMS (Element XR, Thermo Scientific).

### Statistical Analyses

Statistical analyses were performed using GraphPad software (La Jolla, California, USA, www.graphpad.com) to determine the significance of the differences between treatments.

## Results

### Temperature and Light Intensity Conditions for Growth of *S. kawagutii*

Previous laboratory studies on *Symbiodinium* sp. have been conducted using light intensities ranging from 40 to 600 μE m^-2^ s^-1^ and temperatures from 24°C to 34°C (Robison and Warner, 2006; Saragosti et al., 2010; Buxton et al., 2012; McGinty et al., 2012). We conducted preliminary growth experiments with different light intensities (250 or 600 μE m^-2^ s^-1^) and temperatures (23 or 27°C) to ensure that subsequent experiments would be carried out in conditions amenable to *S. kawagutii* (**Supplementary Figure S1**). We found that 600 μE m^-2^ s^-1^ and 27°C represented favorable conditions for *S. kawagutii* growth when Fe, Mn, Cu, Zn, and Ni were present in adequate supply (**Supplementary Figure S1**). These light intensities and temperatures were close to ambient light intensity (640 μE m^-2^ s^-1^) and temperature (25°C) observed in most coral reefs (Hoegh-Guldberg et al., 2007; Brodersen et al., 2014). We therefore used these conditions (hereafter referred to as Control treatment) for subsequent experiments to elucidate the effect of trace metal limitation on *S. kawagutii* growth.

### Influence of Cu, Zn, Mn, and Ni on Growth of *S. kawagutii*

**FIGURE 1:**
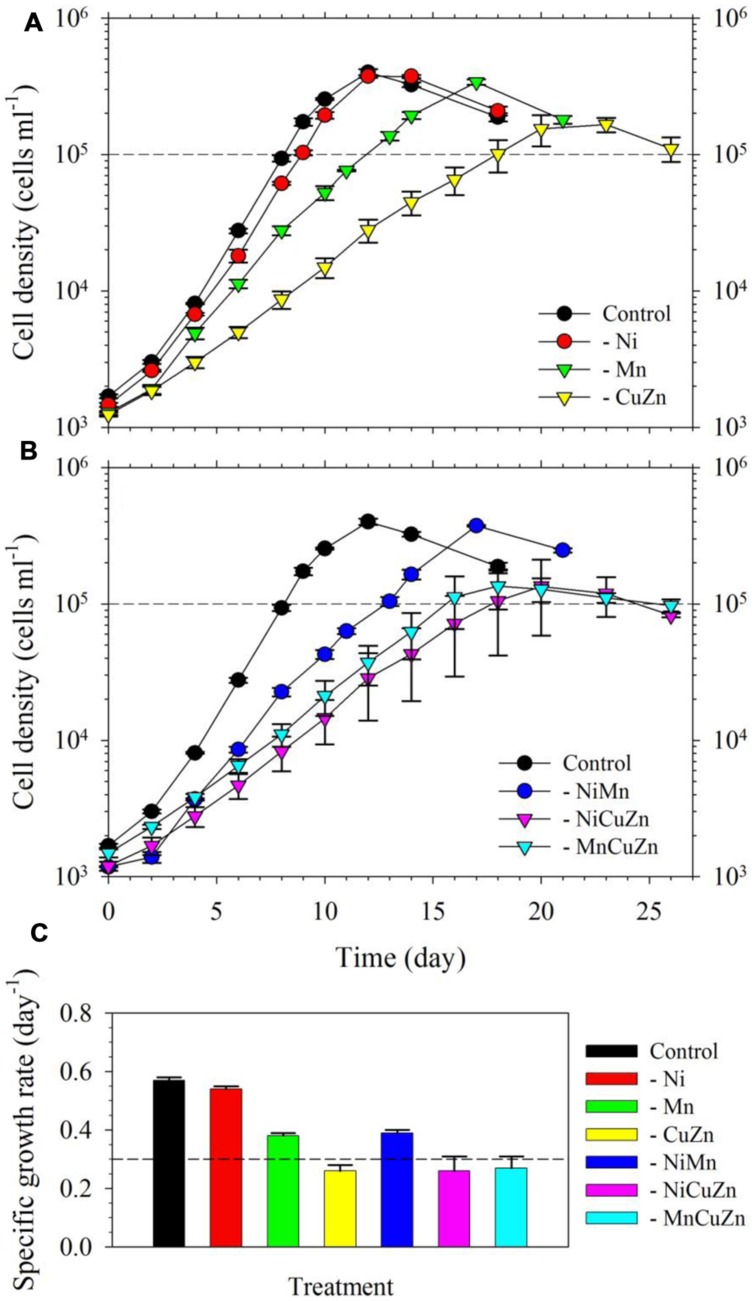
**Growth curves and rates of *Symbiodinium kawagutii* cultures grown in different trace metal availabilities.** **(A)** Growth curves of *S. kawagutii* in culture medium without Ni, Cu/Zn, or Mn. **(B)** Growth profiles when two of Ni, Cu/Zn, and Mn were simultaneously excluded in the growth medium. **(C)** Observed specific growth rates in the different treatments. All treatments were supplied with 2.5 pM Fe′. Error bars represent standard deviations of triplicate culture bottles for each treatment.

We found that *S. kawagutii* growth was affected differently when Ni, Cu/Zn, or Mn was omitted in the growth medium (**Figure 1A**). Among the three treatments, growth conditions without Cu/Zn and Mn resulted in significantly lower growth rates compared to that in the control (*p*-value <0.0001, **Figures 1A,C**). In the treatment without Ni, the growth curve and biomass were not substantially altered but gave slightly, although still significant, lower growth rate relative to that in the control treatment (*p*-value = 0.02). Between low Cu/Zn or low Mn availability, growth of the zooxanthellae was influenced more by Cu/Zn availability as reflected by both lower growth rate and lower maximum biomass (∼1.5 × 10^5^ cells ml^-1^ versus ∼ 3 × 10^5^ cells ml^-1^). Further experiments, wherein pairs among Ni, Cu/Zn, or Mn were left out of the growth medium, validated earlier findings (**Figure 1B**). Low Ni availability, paired with either low Cu/Zn or low Mn, did not lead to significant differences compared to treatments without Cu/Zn or Mn. The treatment where Cu/Zn/Mn were left out resulted in a growth curve and rate comparable to that in the low Cu/Zn treatment. Our results clearly show that Cu, Zn, and Mn are important for *S. kawagutii*, while Ni may not be significant for its growth and its anti-oxidative defense under the growth conditions used.

**FIGURE 2:**
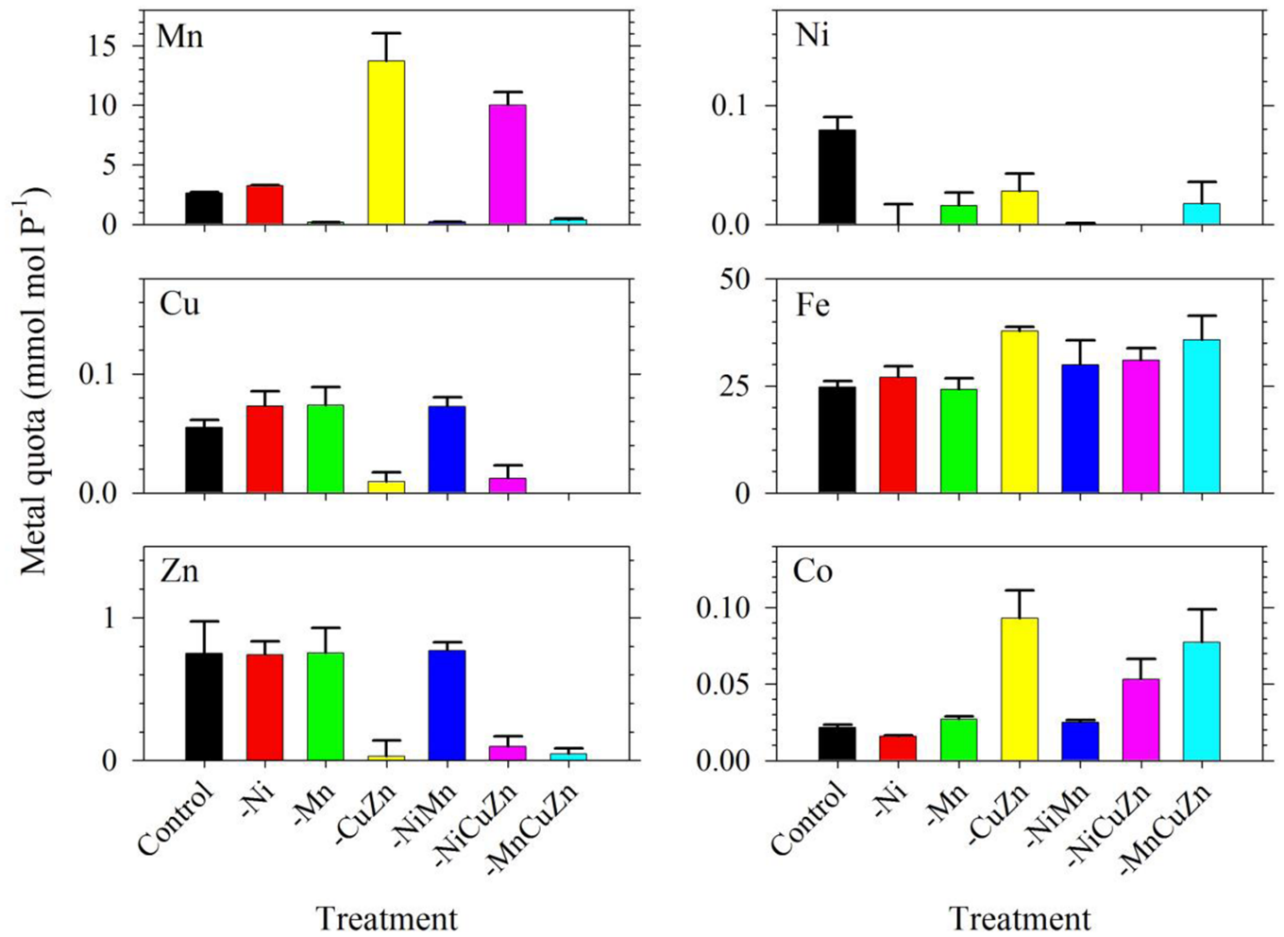
**Intracellular trace metal quotas of *Symbiodinium* cultures subjected to different trace metal availabilities.** The Control treatment was supplied with 67 pM Ni′, 42 nM Mn′, 0.5 pM Cu′, and 125 pM Zn′. A minus sign in a particular treatment means that specific metal or metals were not added to the growth medium. All treatments were supplied with 2.5 pM Fe′. Metal quota was normalized to phosphorus content of the cells as indicator of biomass. Error bars represent standard deviations of triplicate culture bottles for each treatment.

The intracellular metal quotas, normalized against phosphorus as biomass indicator, complemented the observed growth profiles for respective treatments (**Figure 2**). The intracellular Cu, Zn, and Ni quotas were generally consistent with the availability of metals in growth medium. In general, Fe quotas ranged from 25 to 35; Mn quotas ranged from 3 to 14; Zn quotas were close to 0.8; Cu varied from 0.06 to 0.08; and Ni ranged from 0.02 to 0.08, with all values having the unit mmol mol^-1^ P. In the case of Mn, it is noteworthy that intracellular Mn quotas in treatments with low Cu/Zn and low Ni/Cu/Zn were significantly higher compared to quotas in the control and other treatments. Similar to the case of Mn quotas, intracellular Fe and Co quotas also increased slightly in treatments where Cu and Zn were not added. All of these treatments were carried out in media supplied with 2.5 nM of inorganic Fe (Fe′) and 20 pM of inorganic Co (Co′). With these intracellular metal quotas and the observed growth rates presented in **Figure 1**, metal uptake rates estimated by multiplying the intracellular quotas with corresponding growth rates (Sunda, 2012) reveal remarkable differences (**Supplementary Table S1**). Fe uptake rates showed comparable values for the control and low Ni treatments, which were the highest compared to that in all other treatments. Fe uptake rates for low Mn, low Cu/Zn, and low Ni/Cu/Zn treatments were also comparable while rates for low Ni/Mn and low Mn/Cu/Zn treatments were almost equivalent. Consideration of the standard deviation though leads to comparable uptake rates for most of the treatments. Uptake of Mn was higher in low Cu/Zn and low Ni/Cu/Zn treatments suggesting a possible replacement of Mn for either Cu or Zn. Uptake rates for Cu, Zn, and Ni were all consistent with the respective metal availability in the growth medium. We note though that the varying inorganic concentrations of metals we used in this experiment, from 0.5 pM for Cu′ to 42 nM for Mn′, may have significantly influenced the uptake rates.

### Influence of Fe on Growth of *S. kawagutii*

**FIGURE 3:**
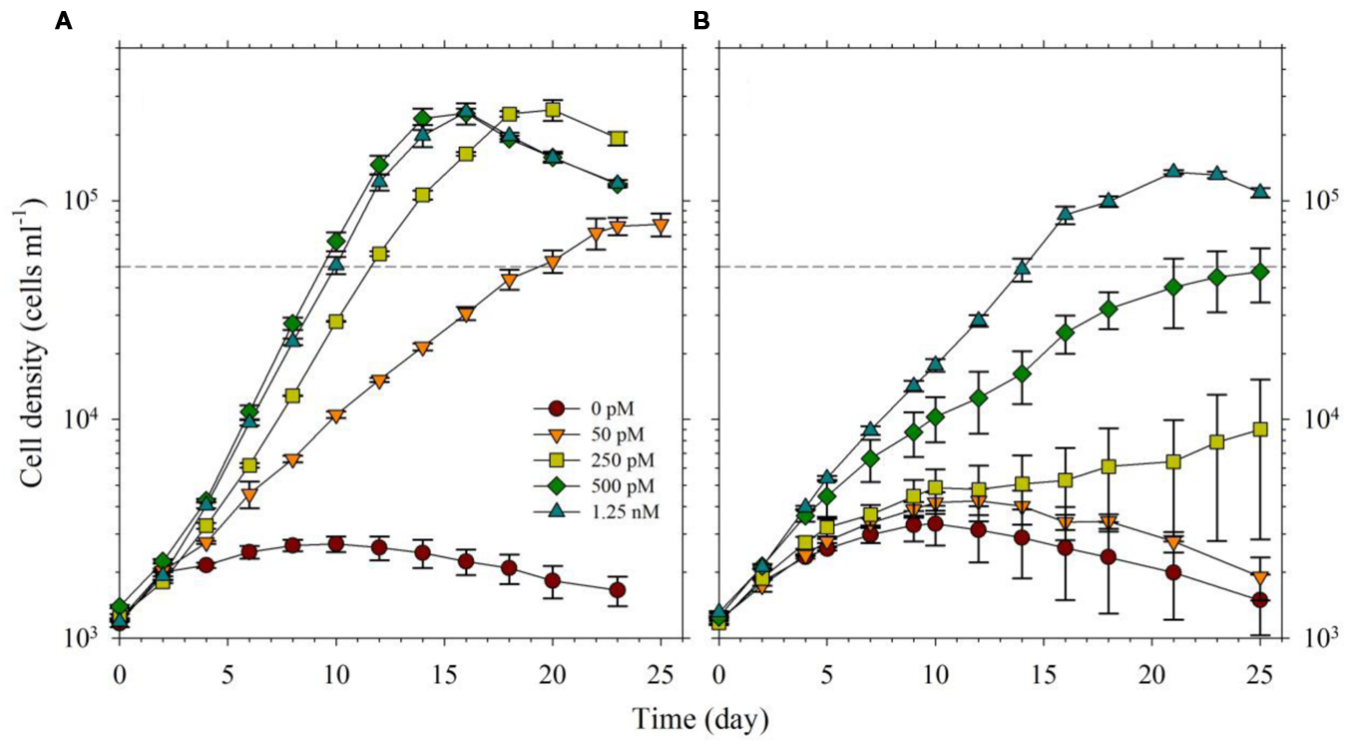
**Growth curves of *Symbiodinium* cultures grown in varying Fe′ concentrations (0, 50, 250, 500, and 1250 pM Fe′).** Effect of Fe availability was studied: **(A)** with 0.5 pM Cu′, 125 pM Zn′, and 42 nM Mn′ in the growth medium, and **(B)** without all of these in the culture medium. Error bars represent standard deviations of triplicate culture bottles for each treatment.

**FIGURE 4:**
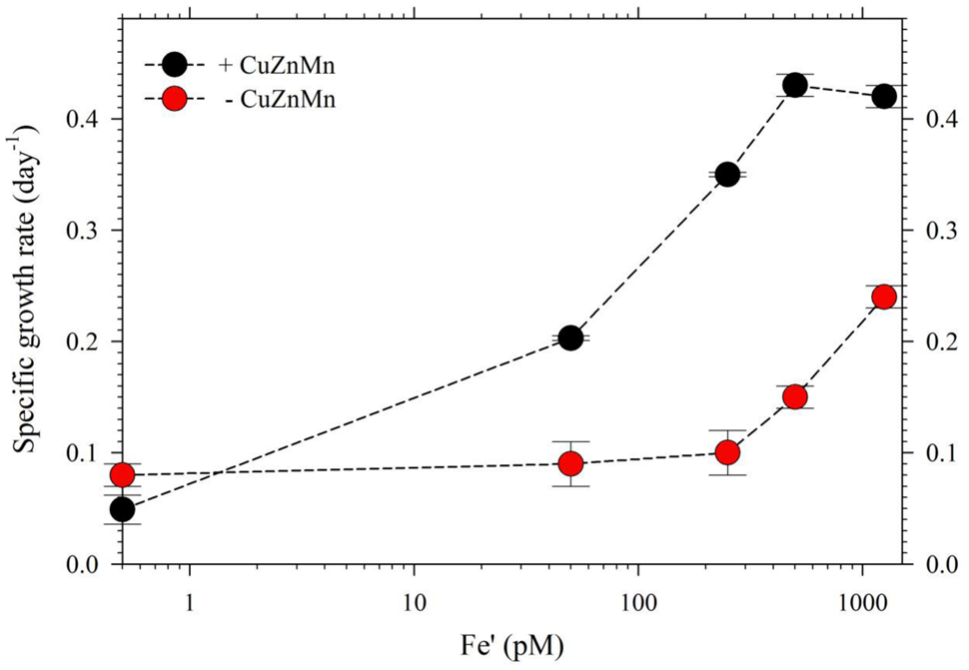
**Growth rates of *S. kawagutii* cultures subjected to varying concentrations of Fe′ (0, 50, 250, 500, and 1250 pM).** Two sets of treatments were carried out: one with 0.5 pM Cu′, 125 pM Zn′, and 42 nM Mn′ supplied and the other without these altogether in the culture medium. Growth rates were calculated while cells were in logarithmic phase of the growth period (days 2–19 depending on the treatment; see **Figure 3**). Error bars represent standard deviations of triplicate culture bottles for each treatment.

For the second part of this study, we acclimatized *S. kawagutii* in culture media with respective Fe′ concentrations, 0, 50, 250, 500, and 1250 pM, prior to conducting actual experiments. The growth curves showed that Fe′ concentrations equivalent to or higher than 50 pM were sufficient to sustain growth of *S. kawagutii* when Cu, Zn, and Mn were added to the medium (**Figure 3A**). However, the maximum biomass of *S. kawagutii* grown in 50 pM Fe′ only reached about 1.0 × 10^5^ cells ml^-1^, which was significantly lower compared to biomass in higher Fe′ treatments. In the set of treatments without Cu, Zn, and Mn, comparison of growth curves showed that *S. kawagutii* required higher Fe availability to sustain growth (**Figure 3B**). For instance, the –Cu/Zn/Mn cultures supplied with Fe at concentrations equal to or higher than 50 pM Fe′ reached lower biomass than in corresponding Fe conditions in the set of treatments with Cu/Zn/Mn. The effect of deficiency of Cu, Zn, and Mn included lower growth rates and lower cell concentration maxima (**Figures 3B** and **4**). The average growth rates calculated from the exponential growth phase revealed that high Fe availability and ample supply of Cu, Zn, and Mn lead to relatively high growth rates (**Figure 4**).

**FIGURE 5:**
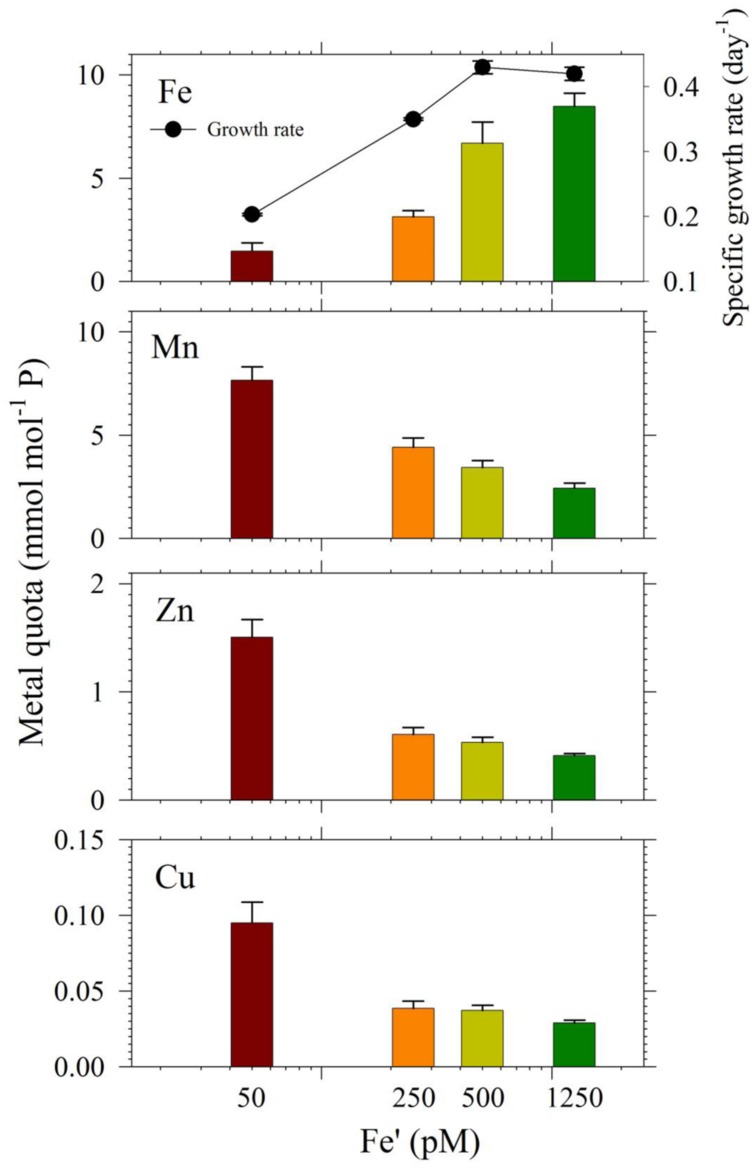
**Intracellular metal quotas, normalized against phosphorus as the biomass indicator of *S. kawagutii* cultures subjected to different Fe′ concentrations (50, 250, 500, and 1250 pM), and with 0.5 pM Cu′, 125 pM Zn′, and 42 nM Mn′.** Error bars represent standard deviations of triplicate culture bottles for each treatment.

In the second experiment, we harvested cells in treatments with Cu/Zn/Mn added for quota determination, except the 0 pM Fe′. While for the set of treatments without the three metals, only cells from the highest Fe′ treatment (1.25 nM Fe′) were harvested for quota determination because the other Fe′ treatments had low cell densities. The intracellular quotas we determined correlated well with observed growth rates for respective treatments (**Figure 5**). Intracellular Fe quotas increased corresponding to increasing Fe supply in the medium. Evaluation of uptake rates showed roughly threefold increases in rates from Fe′ concentrations of 50 to 500 pM but only a slight increase from 500 to 1250 pM Fe′ (**Supplementary Table S2**). We also compared Fe quotas in treatments with 1.25 nM Fe′ from both the with- or without- Cu/Zn/Mn sets. The Fe quota in the 1.25 nM Fe′ treatment with Cu/Zn/Mn added was considerably lower than in corresponding treatment without Cu/Zn/Mn added, which were 8.7 ± 0.7 and 11.6 ± 1.4 mmol mol^-1^ P, respectively. The intracellular quotas of Cu, Zn, and Mn increased with decreasing Fe availability in the medium. However, evaluation of uptake rates from elemental quotas and growth rates showed that Zn and Mn uptake rates were only slightly higher in low Fe′ treatments while Cu uptake rates were comparable in all Fe′ treatments (**Supplementary Table S2**).

## Discussion

Trace metal uptake and intracellular quotas in phytoplankton are a total reflection of both their biochemical demand and extracellular trace metal supplies (Sunda, 2012). In this work, we set out to understand the quantitative importance of trace metals on *S. kawagutii* growth based on growth curves and intracellular quotas. While the trace metals considered in this study are cofactors in various SOD and some other antioxidants (catalase and peroxidase), they are also utilized in many other cellular enzymes and metabolic reactions (Raven et al., 1999; Sunda, 2012; Twining and Baines, 2013). Because bioavailable metal concentrations are crucial and will impact the metal uptake by the organism, the use of artificial or natural seawater with known trace metal concentrations are necessary in studies pertaining to trace metal requirement and limitation. We controlled the bioavailable trace metal concentrations in our culture media by passing the seawater through a chelating resin following the procedures outlined elsewhere (Ho, 2013) and using EDTA to control the inorganic metal concentrations in different treatments. The Fe and Mn concentrations in the traditional L1 medium are extremely high at 12,000 and 900 nM, respectively (Guillard and Hargraves, 1993), in which the bioavailable trace metal concentrations are not defined. We used much lower total dissolved concentrations of Fe and Mn in our experiments, but the metal concentrations we used may still be higher than natural concentrations in the ocean.

In our first experiment, the bioavailable or inorganic concentrations (M′) varied significantly among the metals in the media, with concentrations of 2.5 nM, 42 nM, 125 pM, 0.50 pM, and 67 pM for Fe, Mn, Zn, Cu, and Ni in treatments designed to contain specific metals, respectively. The growth curves and rates obtained in the first experiment clearly indicate that among Mn, Cu/Zn, and Ni treatments, Cu/Zn availability influence *S. kawagutii* growth the most (**Figure 1**). Comparison of growth curves and intracellular quotas for treatments with varying trace metals reveal that metal quotas normalized to P were highest for Fe, followed by Mn, then Zn/Cu, and lowest for Ni (**Figures 1** and **2**). Although bioavailable Mn concentrations in culture media were over an order of magnitude higher than Fe′, Fe quotas were significantly higher than Mn quotas in *S. kawagutii*, showing relatively high Fe requirement in this species. Also, although Ni′ concentration used in this study was about half of the Zn′ concentration available in the medium, Ni quotas were an order of magnitude lower than Zn quotas. **Figure 1** also shows that Ni availability only slightly influenced the growth curves and rates suggesting that Ni may not be as important as the other metals for *Symbiodinium*. Among all metals, Cu′ availability was the lowest and this low availability was also reflected in low Cu quotas observed in the treatments.

Among all treatments, low Cu/Zn treatments resulted in marked differences in terms of maximum biomass and growth rates achieved, either independently or paired with low Ni or low Mn. While we are limited by the research design employed, specifically the coupling of Cu and Zn due to consideration that both metals are present in Cu/Zn-SOD, our results highlight the importance of these two metals in the dinoflagellate. The lower rates and biomass signify that deficiency of either Cu or Zn or the compounded effect of low supply of both metals in the growth medium, result in conditions that were not amenable for *S. kawagutii*. This certainly warrants further studies, because of varied biochemical processes requiring Cu or Zn but not both. Another remarkable observation in the low Cu/Zn treatments would be the elevated Mn, Fe, and Co quotas, which imply that cells increased uptake of these metals to compensate for low Cu/Zn availability. Although there is a possibility that the increase in quotas may be due to relaxed competition for shared divalent metal transporters, it is at least equally likely that the elevated Mn and Fe quotas may have been primarily due to compensatory increase in Mn- and Fe-SOD production (Kustka et al., 2007; Lane et al., 2008). In the case of Co, the higher intracellular quotas may have been due to increase in Co-carbonic anhydrase (Co-CA) to compensate for low Zn-CA (Sunda and Huntsman, 1995). Evaluation of Fe uptake rates in low Cu/Zn and low Ni/Cu/Zn treatments show lower rates compared to that in other treatments (**Supplementary Table S1**). Mn uptake rates for low Cu/Zn and low Ni/Cu/Zn treatments, however, were highest among all treatments. This discrepancy in uptake rates observed in low Cu/Zn and low Ni/Cu/Zn treatments points to differing mechanisms that require more in-depth studies to ascertain what processes caused the elevated Mn, Fe, and Co quotas.

In the experiment with varying Fe′, we observed that Fe′ concentration of 50 pM was sufficient for *S. kawagutii* to achieve high growth rates at conditions of high light intensity, high temperature and with adequate supply of Cu′ (0.50 pM), Zn′ (125 pM) and Mn′ (42 nM; **Figures 3A** and **4**). In the corresponding set without Cu/Zn/Mn, increasing Fe′ concentrations resulted in higher biomass attained by *S. kawagutii*, but all were lower compared to biomass achieved by corresponding Fe treatments in the set with Cu/Zn/Mn (**Figure 3B**). This disparity in observed biomass clearly indicated that the absence of Cu/Zn/Mn in culture medium negatively affected *S. kawagutii* growth. Low concentrations of these metals likely forced the zooxanthellae to rely only on its Fe supply for both its photosynthetic requirements and antioxidant mechanisms by switching to Fe-dependent proteins and enzymes. In the set of treatments where Cu, Zn, and Mn were supplied, growth rates were about two to three times higher than in corresponding Fe′ treatments without these three metals (**Figure 4**). This reduction in growth rates in the absence of Cu/Zn/Mn and in low Fe availability could pose serious consequences to the coral-algal symbiosis when it is subjected to stress factors. Survival of the symbiotic relationship and coral reefs in general depends on the capacity of corals to re-populate itself with *Symbiodinium* in cases when population of the symbiont diminishes (Roth, 2014). Our results show that low Fe concentrations, exacerbated by low supply of other metals, may result in slower growth of the symbiont population leading to reduced cell densities or complete breakdown of the symbiosis. We also observed that in the set of treatments without Cu/Zn/Mn, cells tended to coalesce and form an intricate colony (data not shown). This characteristic tendency to form aggregates has previously been reported in studies on the role of light quality in *Symbiodinium* growth but no reason was provided to explain this behavior (Hennige et al., 2009). We surmise that this observation may be a cellular response to oxidative stress but this merits further investigation.

The intracellular Fe quotas increased with increasing Fe availability in the medium, which generally corresponded with the observed growth rates (**Figure 5**). This Fe quota to growth rate correlation was entirely expected, because of the inherent Fe requirement in important biochemical processes. A closer inspection of the difference in growth rates from 500 to 1250 pM Fe′, however, shows comparable growth rates between treatments indicating that the increase in Fe availability did not necessarily translate to better growth conditions. This demonstrates that 500 pM Fe′ may be the threshold inorganic Fe concentration for *S. kawagutii* to achieve sufficiently high growth rates and biomass in the growth conditions used. A previous study reported much higher inorganic Fe requirement by *Alexandrium tamarense*, a toxic dinoflagellate, which attained a growth rate equivalent to 0.5 day^-1^ in cultures supplied with about 3 nM available Fe (Wells et al., 1991). Comparing the Fe requirement of *S. kawagutii* to other well-studied phytoplankton also reveal that the growth rate reached by this dinoflagellate in medium with 500 pM Fe′ was comparably high. *Thalassiosira pseudonana*, a model coastal diatom species, reached a growth rate of nearly 2 day^-1^ in cultures supplied with ∼800 pM Fe′ while *Thalassiosira weissflogii*, another model coastal diatom species, almost reached a growth rate of 1 day^-1^ in cultures with ∼300 pM Fe′ (Sunda and Huntsman, 1997). The rates achieved by *Prorocentrum minimum* and *Prorocentrum micans* were about 0.5 and 0.25 day^-1^ in Fe′ conditions equivalent to 100 pM, respectively (Sunda and Huntsman, 1997). The experiments on *Thalassiosira* and *Prorocentrum* were conducted using light intensity of 500 μE m^-2^ s^-1^ and this lower intensity may have influenced the Fe requirement owing to enhanced requirement for photosynthetic proteins in low light conditions. Iron utilization by photosynthetic organisms involves complex mechanisms that require them to continuously adapt to low Fe environments as highlighted in recent published works (Morrisey and Bowler, 2012; Lis et al., 2015). Our results show that *S. kawagutii* requires relatively high Fe availability, equivalent to 500 pM Fe′, to attain high growth rates but it may also thrive, albeit with lower growth rate, in conditions with low Fe availability and sufficient supply of Cu, Zn, and Mn.

The intracellular quotas for Cu, Zn, and Mn increased with decreasing Fe′ implying that cells subjected to lower Fe′ status offset the shortage of Fe supply by assimilating greater amounts of Cu, Zn, and Mn (**Figure 5**). Similar to elevated Mn uptake observed in previous experiments, it may be argued that the low Fe supply might have caused the cells to overexpress non-specific divalent metal transporters, which leads to random uptake of any divalent metals (Kustka et al., 2007; Lane et al., 2008). However, it is at least equally probable that the inverse correlation was a result of specific response to low Fe availability, and elevated uptake and assimilation of Cu, Zn, and Mn were needed to compensate for the lower Fe′ availability for essential cellular functions such as Cu/Zn- and Mn-SOD production. Evaluation of uptake rates showed that Mn and Zn uptake rates slightly increased with decreasing Fe′ availability while Cu uptake rates remained comparable in all treatments (**Supplementary Table S2**). These results indicate the possibility of functional complementation among trace metals in *S. kawagutii*, where one can partially replace some of the biochemical functions of another, which is an interesting topic for further study. These results provide fundamental information about trace metal requirements and cellular quotas in *S. kawagutii* and on how the essential trace metals interact with each other to reduce stresses caused by deficiency of some of these metals. This fundamental information is vital for better understanding of the growth requirements of *Symbiodinium* in its native environment.

In nature, trace metal supplies to *Symbiodinium*, along with other nutrients and amount of light, are in part regulated by the coral host essentially because *Symbiodinium* is sheltered in the host endoderm. It follows that *Symbiodinium* may not be able to fully utilize the ambient dissolved trace metals present in seawater surrounding corals. The dissolved Fe concentrations in these regions are typically very low at about 0.61 to 3.5 nM, while dissolved Mn at intermediate salinities normally fall within the range 22 to 242 nM (Roitz et al., 2002; Chase et al., 2005; Tagliabue et al., 2012). Dissolved Cu concentrations may range from as low as 0.03 nM to as high as 33 nM in coastal seawater (Balls, 1985; Bruland et al., 2000; Censi et al., 2006). Total dissolved Zn in coastal waters may be found from about 4.6 nM to about 26 nM (Censi et al., 2006). In addition to low concentrations, metal bioavailability may be controlled by its interaction with natural organic matter rendering it not bioavailable to symbiotic dinoflagellates. However, there are some factors inherent in these regions such as intense light or river discharges that may influence the influx and bioavailability of trace metals. Light intensity may be an important factor to augment Fe bioavailability through photo redox cycling of Fe-ligands (Sunda and Huntsman, 2003). The prevalent metal concentrations coupled with growth information from our results indicate that low-metal availability in its natural habitat subjects *Symbiodinium* to unfavorable conditions that hinder its growth and compromise its capacity to cope with mounting environmental stressors. Future coral ecosystem studies should include the measurement of trace metal concentrations and examination of their relationship with the growth of corals and their symbionts.

The pervasive challenges posed by impending environmental changes, which spans from elevating temperatures to ocean acidification, call for a deeper and wider understanding of factors controlling the growth or sustainability of marine organisms. Our study provides an essential baseline dataset to better understand the role of Fe and other relevant trace metals on the capability of *Symbiodinium* to thrive in conditions of high light intensity and cope with increasing thermal stress. We offer clear evidence of how *S. kawagutii* growth is affected by changes in bioavailability of Fe and other trace metals such as Cu, Zn, and Mn. Natural changes like ocean acidification, increase in light intensity, or temperature may alter predominant chemical processes governing bioavailability of these metals. Thus organisms will have to adapt to a changing ocean. Our results show that *Symbiodinium* may flourish freely in an environment with ample supply of Fe, Cu, Zn, and Mn but its growth will be hampered in an environment lacking these metals. While high Fe availability may compensate for low concentrations of Cu, Zn, and Mn, Fe alone may be able to sustain *Symbiodinium* up to a certain extent. However, it will not manage to support a critical biomass needed for the coral-algal symbiont to withstand a barrage of multiple bleaching stressors. The chemistry and availability of these trace metals, which will be dictated by prevailing conditions in the future global environment, e.g., ocean acidification and resultant decreasing Fe bioavailability (Shi et al., 2010), will therefore have profound impacts on the fate of *Symbiodinium* and hence that of coral reefs.

## Author Contributions

T-YH and IR conceptualized the study; IR and T-YH planned and designed the study; IR and JH carried out the experiments; IR, T-YH, and SL analyzed the data and wrote the paper.

## Conflict of Interest Statement

The authors declare that the research was conducted in the absence of any commercial or financial relationships that could be construed as a potential conflict of interest.The reviewer Brian P. Hedlund and handling Editor declared their shared affiliation, and the handling Editor states that the process nevertheless met the standards of a fair and objective review.
