# Supplementary material for: Effects of Trace Metal Concentrations on the Growth of the Coral Endosymbiont Symbiodinium kawagutii
Source: Front Microbiol. 2016 Feb 8;7:82. doi: 10.3389/fmicb.2016.00082 (PMC4744903; doi:10.3389/fmicb.2016.00082)
Supplement: Supplementary file 1 [file Data_Sheet_1.PDF]

Supplementary Figure and Tables

Effects of trace metal concentrations on the growth of the coral endosymbiont *Symbiodinium kawagutii*

Irene B. Rodriguez[1], Senjie Lin[2,3], Jiaxuan Ho[1,4], and Tung-Yuan Ho[1]*

[1]Research Center for Environmental Changes, Academia Sinica, Taipei, Taiwan

[2]State Key Laboratory of Marine Environmental Science, Xiamen University, Xiamen, China

[3]Department of Marine Sciences, University of Connecticut, Groton, CT, USA

[4]School of Marine Sciences and Engineering, Plymouth University, Devon, UK

*Corresponding author: tyho@gate.sinica.edu.tw

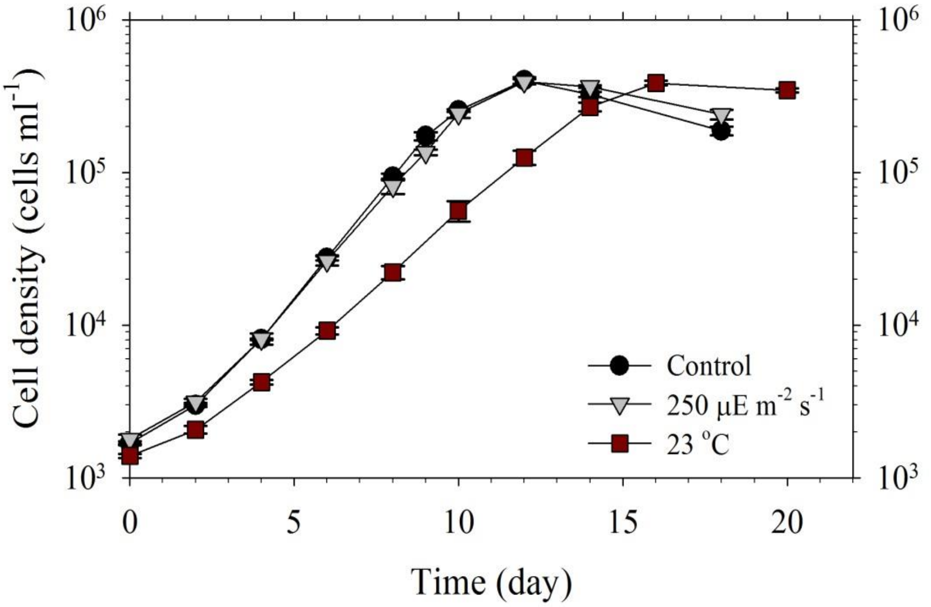

Supplementary Figure 1. **Growth curves of *S. kawagutii* cultures grown in varying light intensities and temperature.** Cultures grown in 600 µE m^{-1} s^{-1} and 27 °C were denoted as the Control treatment. All treatments were supplied with equivalent molar concentrations of trace metals (2.5 nM Fe′, 67 pM Ni′, 0.5 pM Cu′, 125 pM Zn′, and 42 nM Mn′). Error bars represent standard deviation of three replicate culture bottles.

Supplementary Table 1. Uptake rates for different metals by *S. kawagutii* grown in media with varying trace metal inorganic concentrations. The Control treatment was supplied with 67 pM Ni′, 42 nM Mn′, 0.5 pM Cu′, and 125 pM Zn′. A minus sign in a particular treatment means that specific metal or metals were not added in the growth medium. All treatments were supplied with 2.5 pM Fe′. Uptake rates were estimated by multiplying the elemental quotas with corresponding growth rates per treatment.

Treatment	Uptake rate (mmol mol^{-1} P day^{-1})				
	Fe	Mn	Zn	Cu	Ni
Control	14.3 ± 0.9	1.49 ± 0.06	0.41 ± 0.13	0.03 ± 0.00	0.04 ± 0.00
- Ni	14.6 ± 1.5	1.75 ± 0.04	0.33 ± 0.02	0.04 ± 0.01	0.00 ± 0.00
- Mn	9.3 ± 1.0	0.06 ± 0.00	0.22 ± 0.01	0.03 ± 0.01	0.01 ± 0.00
- Cu/Zn	9.9 ± 0.8	3.57 ± 0.66	0.01 ± 0.00	0.00 ± 0.00	0.01 ± 0.00
- Ni/Mn	11.8 ± 2.2	0.08 ± 0.01	0.25 ± 0.02	0.03 ± 0.00	0.00 ± 0.00
- Ni/Cu/Zn	8.2 ± 1.7	2.61 ± 0.58	0.00 ± 0.00	0.00 ± 0.00	0.00 ± 0.01
- Mn/Cu/Zn	11.0 ± 3.9	0.10 ± 0.03	0.00 ± 0.00	0.00 ± 0.00	0.01 ± 0.01

Supplementary Table 2. Uptake rates for different metals by *S. kawagutii* grown in media with varying Fe′concentrations, and with 42 nM Mn′, 0.5 pM Cu′, and 125 pM Zn′. Uptake rates were estimated by multiplying the elemental quotas with corresponding growth rates per treatment.

Fe′ concentration	Uptake rate (mmol mol^{-1} P day^{-1})			
	Fe	Mn	Zn	Cu
50	0.30 ± 0.08	1.55 ± 0.13	0.31 ± 0.09	0.02 ± 0.00
250	1.09 ± 0.01	1.55 ± 0.15	0.21 ± 0.02	0.01 ± 0.00
500	2.88 ± 0.44	1.47 ± 0.15	0.23 ± 0.02	0.02 ± 0.00
1250	3.56 ± 0.29	1.02 ± 0.11	0.17 ± 0.01	0.01 ± 0.00
